# MicroRNA‑124: an emerging therapeutic target in central nervous system disorders

**DOI:** 10.1007/s00221-022-06524-2

**Published:** 2023-03-24

**Authors:** Wen-Hao Zhang, Lian Jiang, Mei Li, Jing Liu

**Affiliations:** 1 Department of Pediatrics, Chinese PLA Medical School/Chinese PLA General Hospital, Beijing, 100095 China; 2 Department of Pediatrics, The 4th Hospital of Hebei Medical University, Shijiazhuang, 050010 China; 3 Department of Neonatology, Maternal and Child Health Hospital of Chaoyang District, Chaoyang District, Beijing, 100020 China

**Keywords:** Central nervous system, NSPCs, miR-124, Brain diseases

## Abstract

The central nervous system (CNS) consists of neuron and non-neuron cells including neural stem/precursor cells (NSPCs), neuroblasts, glia cells (mainly astrocyte, oligodendroglia and microglia), which thereby form a precise and complicated network and exert diverse functions through interactions of numerous bioactive ingredients. MicroRNAs (miRNAs), with small size approximately  ~ 21nt and as well-documented post-transcriptional key regulators of gene expression, are a cluster of evolutionarily conserved endogenous non-coding RNAs. More than 2000 different miRNAs has been discovered till now. MicroRNA-124(miR-124), the most brain-rich microRNA, has been validated to possess important functions in the central nervous system, including neural stem cell proliferation and differentiation, cell fate determination, neuron migration, synapse plasticity and cognition, cell apoptosis etc. According to recent studies, herein, we provide a review of this conversant miR-124 to further understand the potential functions and therapeutic and clinical value in brain diseases.

## Introduction

Neural stem cells (NSCs) are subpopulation cells in embryonic brain and the sub-ventricular zone (SVZ) and the hippocampus of adult brain with the self-renewing can differentiate into a variety of cells (Jäkel and Dimou 2017; Johansson 2003; Adlakha and Saini 2014; Ji et al. 2013; Åkerblom et al. 2012; Papagiannakopoulos and Kosik 2009; Preethi et al. 2012; Wu et al. 2018; Yu et al. 2008; Di et al. 2014; Wang et al. 2017; Huang and Zhang 2018). By symmetric or asymmetric dividing, NSCs can maintain the stem cell pools and give birth to neurons and gliocytes to establish or integrate into brain tissue (Fei et al. 2014; Kang and Reichert 2015). Nowadays it is demonstrated that NSCs are involved with both development defects and postnatal diseases such as microcephaly, stroke and neurodegenerative diseases (Liu et al. 2013a, b; Pollock et al. 2014; Sheinerman and Umansky 2013).

MicroRNAs (MiRNAs) are short single-stranded non-coding RNAs and proven to take part in physiological and pathological processes in central nervous system (CNS), indicating the great importance of miRNAs in CNS (Pourrajab et al 2014; Sun and Shi 2015). By binding to the 3`UTRs of target mRNA, miRNA can induce the mRNA instability or degradation, and impair the protein synthesis through perfect or imperfect pairing with seed regions. Numerous researches affirmed that single miRNAs could target hundreds of mRNAs, contrarily, single mRNAs could also be regulated by different miRNAs. Thus, through the miRNAs-mRNAs interactions, a comprehensive co-operative network are formed at the post-transcriptional level (Christensen and Schratt 2009; Sun et al. 2012).

MiR-124 was first discovered almost 2 decades ago in mice study by Lagos-Quintana and is a strictly conserved in both species and nucleotide sequences. As a brain-rich miRNA, MiR-124 is about 25–50% of total miRNAs in brain and preferentially expresses in neurons (Lagos-Quintana et al. 2002). Further researches demonstrated that miR-124 was transcribed by three loci in human and mice while in nematode just one. So nematode is regarded as an ideal model for the function study of miR-124 (Sun et al. 2012). Current evidence shows that miR-124 is versatile in many aspects in the brain, such as cell proliferation and differentiation, migration, memory formation, cell apoptosis, and brain degenerative diseases (Di et al. 2014; Higuchi et al. 2016; Åkerblom and Jakobsson 2013; Zhang et al. 2015; Vo et al. 2010; Saraiva et al. 2017). In the human and mouse genomes, miR-124 precursors are derived from three independent genes: miR-124-1, miR-124-2, and miR-124-3 (Neo et al. 2014). miR-124 precursors are highly expressed in neurons and have been increasingly reported as a miRNA with antitumor activity (Sanuki and Yamamura 2021). The mature miR‐124 generation process is quite complex. The gene of miR-124 is transcribed into primary miR-124 (pri-mir-124) in the nucleus under the action of RNA polymerase II; it was transported from nucleus to cytoplasm through transporter exportin-5 and cut into about 21 NT double stranded RNA molecules by nuclease cutter; one of the strands is degraded by helicase to become mature miRNA. The polypyrimidine tract-binding protein (PTBP1) binds pri-miR-124-1directly represses miR-124 expression and blocks DROSHA cleavage in the nucleus (Yeom et al. 2019). Long noncoding ribonucleic acids (lncRNAs) also regulate miR-1–miR-124 signal pathway and play significant roles in multiple fundamental biological processes. Long noncoding RNA (lncRNA) NEAT1 was demonstrated to suppress miR-124 expression by direct interaction in NPC cells (Cheng and Guo 2017). lncRNA metastasis-associated lung adenocarcinoma transcript 1 (MALAT1) could directly bind to miR-124-3p and inhibit miR-124-3p expression, which involved the depression of sphingosine kinase 1 (SphK1) (Liu et al. 2021). miR-124 that directly promotes the expression of its target genes is determined by the binding of both AGO and a neuron-enriched RNA-binding protein, ELAVL3, to target transcripts (Lu et al. 2021). Herein we further elucidate the diverse functions of miR-124 and provide the basis for clinical treatment and scientific research.

## Brief introduction of miRNA biogenesis

**Fig. 1:**
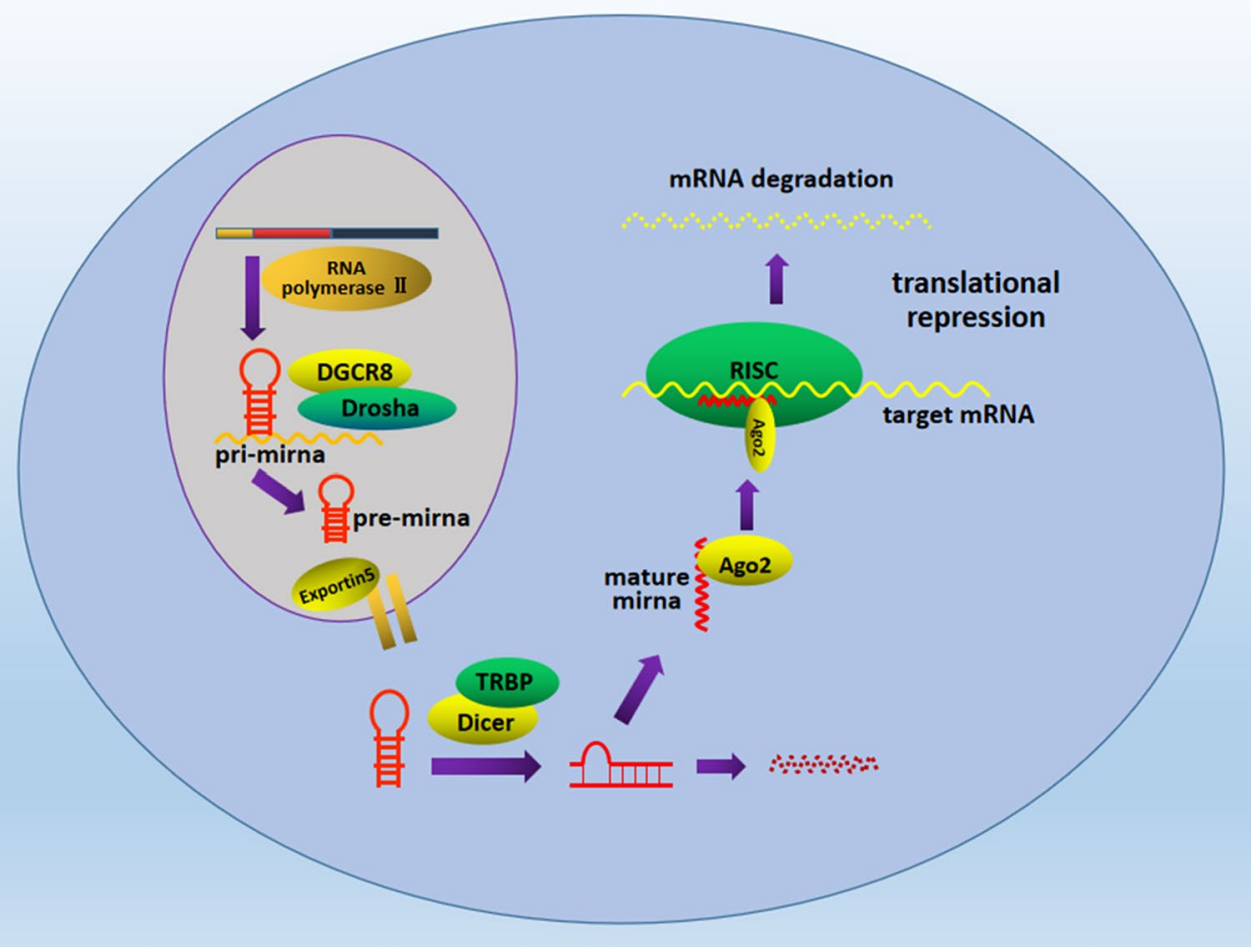
The classical biogenesis of MiRNA and its mechanism of inhibiting protein expression

Most miRNAs are generated in a canonical pathway (Liu et al. 2015). miRNAs can be transcribed as single or clustered from miRNA coding gene introns or exons. In the nucleus, miRNAs are transcribed into pri-miRNAs with stem-loop structure by RNA polymerase II, and the pri-miRNAs are cleaved by the RNAse III enzyme Droshato form pre-miRNAs, which are then exported to the cytoplasm by Exportin-5 and its co-factor. In cytoplasm, another RNAse III enzyme Dicer can recognize pre-miRNAs and make a further cleaving to generate duplex miRNAs: a mature miRNA and a passenger strand. The mature miRNA is incorporated into the RNA-induced silencing complex (RISC) to its target mRNAs to exert the post-transcriptional repress of gene expression. But there are also non-canonical pathway which can bypass Drosha or Dicer (Fig. 1) (Katz et al. 2016; Lee 2013).

## MiR124 in proliferation and differentiation of neural stem/precursor cells (NSPCs)

Lin-4 and let-7 were the first identified miRNAs by Lee and Reinhart et al., which are demonstrated to regulate larval development in *Caenorhabditis elegans* (Sempere et al. 2004). So the crucial function of miRNAs in the brain development emerges to our sight. MiR-124 as the most brain-enriched miRNA is validated to participate in the process of neural stem cell proliferation and differentiation. During the past decade, the main function of miR-124 has been shown that is to restrain NSPC proliferation and promote neuron differentiation by targeting Scp1, Sox9, Cdc42, RE1, and Jag1 in mice*,* however laminin γ1 and integrin β1 in chicken (Mokabber et al. 2018; Visvanathan et al. 2007; Franke et al. 2013; Doeppner et al. 2013; Liu et al. 2011; Kerek et al. 2013). It is validated that folic acid deficiency leads to reduced proliferation of neural precursor cells, and this effect may induce by miR-124. Kerek et al. have found that miR-124 is significantly upregulated in Wistar rats embryo with a methyl donor deficiency (MDD) diet. Overexpressed miR-124 may stimulate NSPCs premature differentiation by repressing the STAT3 (Yang et al. 2019). Recently, Yang et al. has revealed that miR-124 could induce microglia M2 polarization and constrain TLR4 to promote neurogenesis after traumatic brain injury (Yang et al. 2017). In a stroke model, miR-124 loaded in viral-induced exosome has been shown to enlace the cortical neurogenesis (Liu et al. 2011). And recently miR-124 has been demonstrated to promote hair follicle stem cells (HFSCs) differentiating into neurons by directly down-regulating Ptbp1 and Sox9 (Mokabber et al. 2018). Recent studies have shown that miR‑124 promoted the neuronal differentiation of and neurite outgrowth in mouse inner ear NSCs, and that the changes in the expression of tropomyosin receptor kinase B (TrkB) and cell division control protein 42 homolog (Cdc42).

But opposite points still exist. Liu et al. found that miR-124 boosted the cell proliferation and inhibited the cell differentiation at optic vesicle stage earlier than optic cup stage in *Xenopus* during eye development through downregulating theNeuroD1, a proneural marker (Jiao et al. 2018). Lately, Jiao et al. has revealed that miR-124 is involved in both proliferation and differentiation of neuronal stem cells through activating the wnt/β-catenin pathways and inactivating the Notch pathway by negatively regulation of disheveled binding antagonist of β-catenin 1 (DACT1) and Delta-like 4 (DLL4) respectively in murine embryo with 13.5 days (Chen et al. 2011).

In summary, these conclusions which are seemingly contradictory may be the result of the differences either selection of test subjects or the different periods of subjects when tested.

## MiR-124 in neuronal fate decision

Following the differentiation of neural precursor cells, the pluripotent neural precursor cells (NPCs) are switched to single functional neurons accompanied with the inhibition of large scale of non-neural genes and the open expression of neuronal genes (Makeyev et al. 2007). MiR-124, together with its targeting mRNAs and the downstream transcripts, is thought to be pivotal for the transition from non-neuron cells to neurons.

It is worth to point that Eugene et al. have demonstrated that PTBP1 and PTBP2 are both regulated by miR-124, but PTBP1 carries six binding sites on its 3`UTRs contrast to PTBP2 just only one site, thus resulted in better binding to miR-124 and accumulating the PTBP2 expression as a trigger during non-neuron to neuron switch (Yeom et al. 2019). Subsequent studies have validated novel additional functions of PTBP1 in NPCs, where it may constrain or reinforce the targeting of miR-124 by competitive combination or transform the structure of the target mRNAs and reversely inhibiting the maturation of miR-124 by binding to primary form (pri-miR-124) both in vivo and in vitro (Arvanitis et al. 2010) and come to a new conclusion that it is the depression of miR-124 drive the fate decision of non-neuronal cells.

It is reported that Ephrin-B1, a member of ephrin family, which related to lineage restriction, is vital to maintain the fate of NPCs. Dina et al. demonstrated that miR-124 repressed the expression of Ephrin-B1 by directly binding to 3`UTRs to promote differentiation, but, in turn, miR-124 was downregulated in Ephrin-B1-experssioned NPCs. Thus, a feedback loop was formed to regulate the NPCs fate in developing brain (Lim et al. 2005).

It has already been confirmed that miR-124 converts some non-neural stem cells or adult soma cells into neural differentiation (Tang et al. 2013; Zhou et al. 2012), which ensures the crucial role of miR-124 in controlling fate decision of NSPCs. It has been proved that miR-124 promotes the bone marrow mesenchymal stem cells (BMMSCs) to differentiate into neurogenic cells in spinal cord injury model by regulating the PTBP1 and PTBP2 (Chen et al. 2011). Similarly, Dong et al. have validated that miR-124 is upregulated by Ginsenoside Rg1 with a dose-dependent manner to repress the expression of SCP1 and perform the adipose-derived stem cells (ADSCs) neural differentiation. Ambasudhan et al. have affirmed that miR-124 together with MYT1L and BRN2 reprograms dermal fibroblasts to neurons through certain culture condition (Dong et al. 2017).

It is reported that small C-terminal domain phosphatase 1(SCP1) has anti-neural function during development and miR-124 suppresses SCP1 expression and induces neurogenesis (Visvanathan et al. 2007). Cao et al. have affirmed that miR-124 together with laminin gamma 1 and integrin beta1inhibited in neurons (Cao et al. 2007). Suzuki et al. have affirmed that miR-124 reduced the number of SOX9- and GS-positive cells and increased that of TUBB3-positive cells and high-level miRNA overexpression is essential for directing cell fate by miR-124 interference (Suzuki et al. 2020). These studies suggesting that different amounts of miR-124 expression led to different outcomes in fate determination.

In conclusion, miR-124 is not only closely involved in the fate decision of NSPCs, but also has the ability to convert different kinds of cells into neurons. Therefore, miR-124 is a key player in cell fate.

## MiR-124 in neuronal migration

As known to all, the cerebral cortex consists of six layers of neurons, each of which has different cell morphology. After differentiation, these different cells need to migrate to the different layers of cerebral cortex under tight regulation to integrate with other cells into a complete and complex functional network. Current studies have shown that neuronal migration is mainly related to the dynamic changes of cytoskeleton proteins (Heng et al. 2010; Volvert et al. 2014).

Volvert et al. discovered that the specific blocking of the expression of miR-124 and miR-22 in the developing cerebral cortex hindered the migration of projection neurons to the cortical plate (CP) and led to accumulated multipolar neurons resulting from decreased bipolar neurons. Further study has uncovered that both miR-124 and miR-22 with almost the same efficiency repress the expression of CoREST by binding the 3`UTRs, a transcriptional inhibitor of Doublecortin (DCX) which is a type of microtubule-associated protein recognized as immature neuron marker. Along with the downregulating of CoREST by miR-124 and miR-22, the accumulated DCX contributes to the migration of projected neurons (Volvert et al. 2014).

Recently, another research has proved that splicing factor proline–glutamine rich (SFPQ) is a component of CoREST/LSD1 complex, and inhibition of SFPQ impedes radial migration of newborn pyramidal neurons during the developing cortex. So, it is possible that when miR-124 degrades Corset, SFPQ dissociates from the CoREST/LSD1 complex and participates in the radial migration of newborn neurons (Saud et al. 2016).

Zhang et al. have found that in a Dicer1 knockout model, Radial glia (RG), a subtype of neural progenitor cells, acting as the main contributor to the radial migration for neurons, presents delayed morphologic transformation, and this change is conducted by the activation of Notch pathway via elevated Jag2, while Jag2 is validated as a target of miR-124. Moreover, several studies demonstrated that the knockout of Dicer1 resulted in the elimination of numerous miRNAs including miR-124. So here we can infer that in the absence of Dicer1, it is the unusual expression of miR-124 that leads to the abnormal increase of Jag2 expression, which activates the Notch signaling pathway, which leads to abnormal morphologic transformation of RG cells and therefore impacts the radial migration of neurons (Zhang et al. 2015).

As mentioned forehead, the correct expression of miR-124 is proved to be very important to the physiological radical migration of neural cells to different CPs, but in pathological state (e.g., gliomas), the dysregulation of miR-124 may have different effects on cell migration. Compared with the expression of miR-124 and circadian gene CLOCK in normal brains and glioma patients, Li et al. have found that the expression of circadian gene CLOCK is abnormally elevated in glioma patients, while significantly lower expression of miR-124 than that of the normal brain. Further studies showed that miR-124 could directly regulate the expression of CLOCK by binding to 3`UTRs, and it was precisely because of the abnormal expression of miR-124 that the expression of circadian rhythm gene CLOCK was abnormally elevated, which in turn enhanced the activity of NF-κB and therefore promoted the migration of glioma cells (Li et al. 2013). Similarly, both in glioma specimens and glioma cell lines, the expression of miR-124 was also down-regulated, while the direct regulatory target genes, such as the calcium protease subunit 1 (Capn 4) (Cai et al. 2015), the fos-related antigen 2 (Fra-2) (Luo et al. 2018), and ephrin-A receptor 2 (EphA2) (Wu et al. 2018) were aberrantly highly expressed, which could enhance the migration ability of the glioma cells.

## MiR-124 in neural apoptosis

Apoptosis, also known as programmed cell death, is an important biochemical mechanism in organisms. Normal process of apoptosis is indispensable to maintain normal physiological function. Either little or worse apoptosis can lead to a variety of diseases including organ dysplasia, organ dysfunction, neurodegenerative diseases, ischemic injury, autoimmune diseases and cancer (Elmore 2007; D’Arcy 2019). MiR-124 expression is known to be reduced in many cancer cells. Recently, numerous studies have shown that miR-124 can promote apoptosis in a variety of tumors (Sanuki and Yamamura 2021).The expression of miR-124 enhanced apoptosis by regulating the expression of TET1 and TET2 in colon cancer HT29 cells (Zhou 2021). MiR-124 in the nervous system is also closely related to apoptosis. It was reported that miR-124a is required for the prevention of apoptosis in the developing retina and proper axonal development of hippocampal neurons, as it represses Lhx2 translation (Sanuki et al. 2021).

In a congenital hypothyroidism model, it was found that neonatal rats with hypothyroidism showed obvious growth retardation, meanwhile the level of miR-124 in brain was significantly lower than that in normal control group, and the TUNEL-positive cells in hippocampal were significantly higher, suggesting that the decrease of miR-124 was related to the increase of apoptotic cells. The increment of apoptotic neurons could be rescued by supplementation of miR-124 mimics. Further studies confirmed that it could be the possible mechanism that miR-124 protected neurons from apoptosis by decreasing the Bax and Caspase-3 expression and increasing Bcl-2 expression in hypothyroidism (Shao et al. 2015).

The study of epileptic seizures induced by kainic acid showed that the expression of miR-124 and miR-137 in hippocampal NSPCs treated with kainic acid was up-regulated, and the coordination of miR-124 and miR-137 could inhibit the expression of BCL2L13, a pro-apoptotic protein, reduces the expression of Caspase-3 and shows a result of alleviating the apoptosis mediated by Caspase-3. Therefore, the synergistic effect of miR-124 and miR-137 in the epileptic seizure model induced by kainic acid could reduce the apoptosis of the NSPCs through BCL2L13/Caspase-3 pathway (Schouten et al. 2015).

A methyl donor deficiency (MDD) model demonstrated that the expression of miR-124 in the fetal rat brain and in vitro cultured hippocampal NSPCs was up-regulated and the expression of the Stat3 protein was significantly reduced by interacting with 3, UTRs, the proteins of downstream genes Bcl-2 and Bcl-xL were subsequently reduced, thus resulting in promotion of cell apoptosis. In contrast, the silence of miR-124 could at least partially reduce the apoptosis of the NSPCs.

In addition, it was reported that SPRY1 could reduce the production of neurotrophin (BDNF and FGF) by repressing the ERK/CREB signal pathway, and increase the sensitivity of mature neurons to glutamic toxicity, which leads to neuronal death. As a verified target gene, miR-124 and miR-132directly inhibited the expression of SPRY1, but the coordination of miR-124 and miR-132, rather than single miR-124 or miR-132, could better reduced the sensitivity of neurons to glutamate, promote the survival of the neurons under the pathological conditions (Gu et al. 2018).

## MiR-124 in neuronal ischemic reperfusion injury and repairment

Cerebral ischemia/reperfusion injury may occur at any stages of the brain, maybe neonatal or adult, and it`s a complex and finely regulated event involving the repair of neurons after injury, apoptosis, differentiation, migration, neurogenesis and integration of newborn neurons. The levels of a series of brain-specific miRNAs have confirmed that significant changes during the event, which indicates that miRNAs play an important role in this process. It is notable that miR-124 is the star miRNA of such researches. Different or even opposite results make us confused about the role of miR-124 in the process of brain hypoxia/reperfusion injury, despite the mainstream results are that miR-124 is beneficial to ischemia/reperfusion injury (Di et al. 2014; Liu et al. 2013a, b; Sun et al. 2015; Martinez and Peplow 2017).

The report confirmed that miR-124 did not give benefits to ischemia–reperfusion injury by Liu et al. in 2013. They found that over-expression of miR-124 could lead to a slight increase in the volume of infarction without a significant difference. On the contrary, knockdown of miR-124 significantly reduced the volume of infarction. Further studies showed that miR-124 could directly degrade ankyrin-repeat-, SH3-domain- and proline-rich-region-containing proteins (ASPPs) protein (members of p53 family inhibitory proteins) and promote the apoptosis of neurons injured in the brain during infarction (Liu et al. 2013a, b). Weng et al. found that the mi-R124 level increased significantly after infarction 6 h, and lasted for at least 48 h in the plasma of the middle cerebral artery occlusion (MACO) rats. But further analysis revealed that the miR-124 level was not associated with the infarction size. So, the plasma miR-124 may merely be considered to have qualification as an early-biomarker for infarction (Weng et al. 2011).

But other studies have obtained different conclusions, and considered that miR-124 is beneficial to the repair of ischemia–reperfusion injury. At the same time, Doeppner et al.'s research suggested that high expression of miR-124 after ischemia–reperfusion injury in the brain was beneficial to the nervous system. The results showed that ectopic expression of miR-124 could significantly reduce the neuronal death and infarction focus and improve the motor and cognitive function in MACO rats. The mechanism that miR-124 directly degraded the mRNA expression of deubiquitinate Usp14 and decreased the expression of transcription inhibitor RE1 silencing transcription factor (REST), thus presenting the neuroprotective effect at least 4–7 weeks (Doeppner et al. 2013). Zhu et al. found that the expression of miR-124 was down-regulated in the cerebral infarction of MACO rats, and the protein expression of direct target gene Ku70, which is a DNA repair protein and can block apoptosis mediated by Bax, was significantly increased. The introduction of miR-124 inhibitor into the infarction by intracerebroventricular (ICV) infusion could significantly increase the expression of Ku70, reduce the apoptosis of nerve cells and the volume of infarction and improve the neurological score. Therefore, this study showed that the down-regulation of mir-124 was a neuroprotective mechanism (Zhu et al. 2014).

In addition, some researchers believed that miR-124 contributed little to the repairment. In 2017, Yang et al. found that exosomes of miR-124 modified with rabies virus glycoprotein could be accurately transported to the infarction area and promoted the neurogenesis, but it did not refer to the subsequent influence on neuronal apoptosis and infarction volume (Yang et al. 2017).

The controversial roles of miR-124 in ischemia–reperfusion injury and repairment are still inexplicable, and the possible reasons may be as follows: 1. Different quality of the experimental animal; 2. Different embodiment of the animal model; 3. Observation time after treatment is not consistent.

## MiR-124 in synapse formation and plastics

Numerous important functions of the brain, such as memory, emotion and movement, can be achieved via neural networks. The establishment of neural network cannot be separated from the close relationship with neurons, and the main participants and functional implementers of neural networks are the specific cellular substructure of neurons-neurites, which can be roughly divided into axons and dendrites. With the help of these processes, each neuron can connect with numbers of neurons for information transmission, thus constructing a large and sophisticated neural network. MiRNAs are important participants in neural networks and play an irreplaceable role in neurite formation, plasticity, and cognitive function (Vo et al. 2010; Bredy et al. 2011; Olde et al. 2012). As the most abundant MiRNAs in the brain, miR-124 plays versatile roles in the neural networks, including neurite sprouting, elongation, morphological changes and dendritic spine density changes (Schouten et al. 2013; Hu and Li 2017). In experimental animals, it was found that if miR-124 expression was low or absent in the brain, it would lead to abnormal brain morphology, abnormal cognitive ability and so on (Tuoc et al. 2014).

Early studies found that in the miR-124 knockout mouse model, the Lhx2 protein expression was higher than controls, and caused excessive mossy fiber sprouting and abnormal long axons of the hippocampal neurons. While in wild type mice, miR-124 could directly degrade the lhx2 mRNA in the dentate gyrus to give a suitable level of Lhx2 protein, which was recognized beneficial to the normal development of the axons of the hippocampal neurons (Sanuki et al. 2011). RhoGTPase family members have been validated to be indispensable for neuronal neurite growth. MiR-124 has been confirmed to be highly expressed in nascent olfactory bulb neurons, and its over-expression in root mean squared (RMS) enhances dendritic morphogenesis and spinal density and promotes neurite growth, which is illustrated by inhibition of Rock1, RhoG and Rap2a expression in neonatal olfactory bulb via miR-124 (Franke et al. 2013; Xue et al. 2016). Moreover, studies have confirmed that miR-124 could restrainCdc42 or Rac1, resulting in cytoskeleton adjustments and promoting neurite growth during cortical neuronal differentiation (Yu et al. 2008; Franke et al. 2013). Oxysterol-binding protein expression (OSBP) was confirmed a gradually decreased expression pattern and acted as a direct target of miR-124 during normal brain development by Gu et al. Further studies have shown that both over-expression OSBP and inhibition of miR-124 could inhibit the growth and extension of neurons in the cerebral cortex of mice (Gu et al. 2015). The histone deacetylases (HDACs) were considered as an important regulator for synaptic plasticity and axon regeneration. Recently, Gu et al. found that HDAC5 could directly bind to MEF2C and inhibit the activity of myocyte enhancer factor 2C (MEF2C) which has been proved to be closely related to neurogenesis and can enhance the expression of N6-methyladenosine (M6a), a protein related to the germination and plasticity of neurites. In their subsequent studies, HDAC5 was found to be the direct target gene of both miR-124 and miR-9. The synergistic effect of miR-124 and miR-9 made an optimal inhibition of HDAC5, thereby it could regulate the activity of MEF2C-M6a pathway and promote the axonal development in primary neurons in mice (Gu et al. 2018). Recently, the role of miR-124 in normal brain function has been confirmed. MiR-124-1 ± mice showed impaired prepulse inhibition (PPI) and the expression level of Drd2 increased in prefrontal cortex (PFC). miR-124 dosage modulated PFC function through repressing the Drd2 pathway and played a critical role in normal PFC function (Kozuka et al. 2019).

## MiR-124 in cognition

Except for the function in synapse formation, miR-124 was also validated to play irreplaceable roles in cognition, feeling, learning, memory and emotion through the plasticity of synapses (Yu et al. 2008; Rajasethupathy et al. 2009; Michely et al. 2017). Malmevik et al. acquired the stable inhibition of miR-124 by intra-hippocampal injection of miR-124 inhibitors and found that more than 100 pathways including synapse plasticity were up-regulated (Malmevik et al. 2015). A water maze test showed that mice inhibited miR-124 expression could obtain better spatial memory and working memory (Dutta et al. 2013). Another study found that physical environmental enrichment could improve learning and memory ability in rats by up-regulating pre-miR-124 expression (Brenes et al. 2015). Zhao et al. found that resveratrol (RSV) via intraventricular injection improved long-term memory in older mice. The mechanism might be achieved by reducing miR-134 and miR-124 expression which in turn leaded to up-regulation of CREB-BDNF signaling pathways closely related to memory formation (Zhao et al. 2013). Lu et al. confirmed the existence of a ubiquitous single nucleotide polymorphism (SNP) in the pri-miR-124, named as 531,564, and detected that pri-miR-124 carrying C/G genotypes could generate more miR-124. Giraldo et al. found that aggressive behavior in humans was associated with this SNP. AQ-R-S scale assessment showed that people carrying C/G genotypes were less aggressive than those carrying both G/G and genotypes in a Colombian sample (González-Giraldo et al. 2015). Higuchi et al. found that depressive behavior changes in animals subjected to chronic ultra-mild stress, and reduced miR-124 expression in the hippocampus. MiR-124 over-expression via transfection made depressed animals acquiring an improved resilience for chronic unpredicted mild stress (CUMS), while inhibition of miR-124 made animals more prone to depression. Moreover, further studies have found that miR-124 may affect animal consciousness by targeting regulatory HDAC4/5 and glycogen synthesis kinase 3β (GSK-3β) signaling pathways (Higuchi et al. 2016).

Characteristics of Gulf War disease (GWI) found in the 1990s included attention loss, learning and memory difficulties and depression. Pierce et al. established GWI model and found up-regulation of miR-124-3p and miR-29b-3p expression in rat hippocampus. Subsequent researches have demonstrated that intracerebroventricular miR-124 antisense oligonucleotide injection leads to down-regulated miR-124 expression and up-regulated a variety of proteins closely related to cognitive dysfunction such as neurogenesis, memory, anxiety disorder, depression, which have been identified as miR-124 targets to improve GWI cognitive impairment (Pierce et al. 2016; Laferriere et al. 2019).

## MiR-124 in degenerative diseases of the nervous system

Degenerative diseases of the nervous system are a group of diseases mainly characterized by structural changes and loss of function of neurons caused by factors such as abnormal protein synthesis, incorrect modification and enrichment. The incidence increases with age and has a certain genetic predisposition. The most common clinical neurodegenerative diseases are Alzheimer's disease (AD), Parkinson's disease (PD) and Huntington's disease (HD) (Saraiva et al. 2017; Juźwik et al. 2019).

**Fig. 2:**
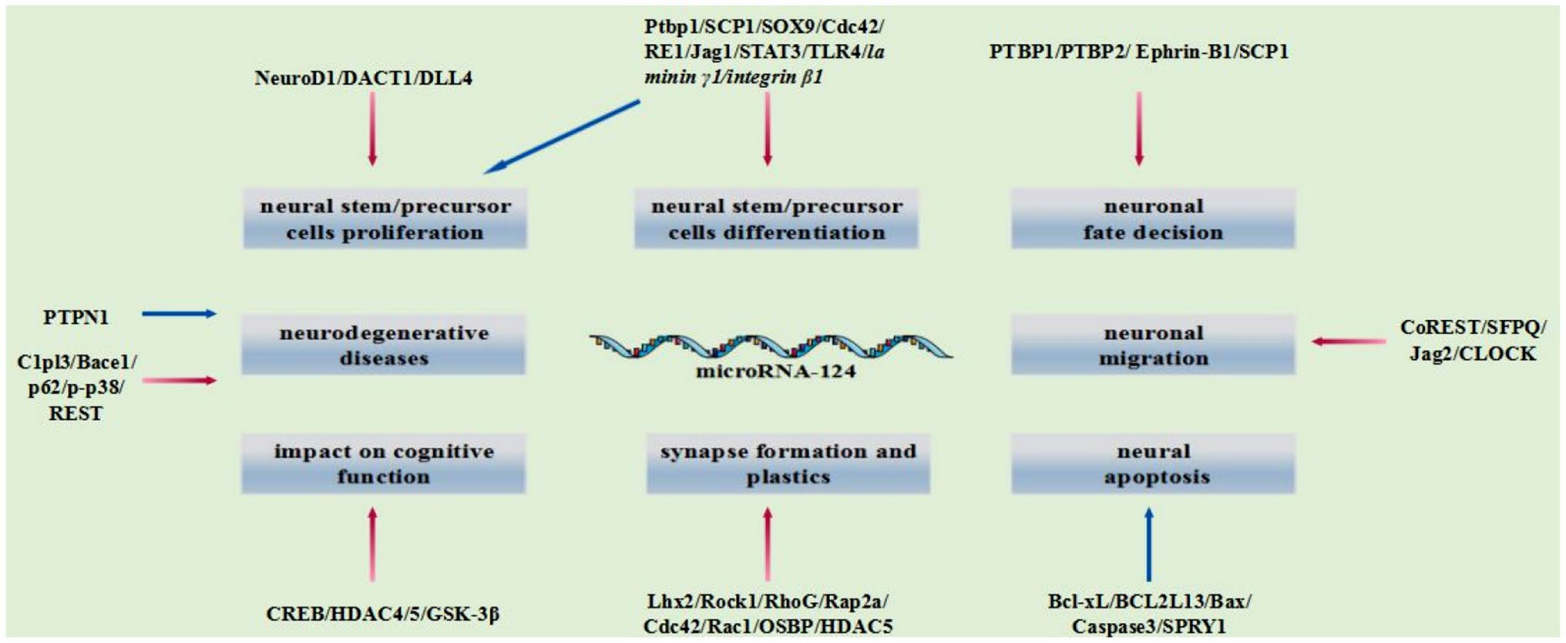
General functions of microRNA-124 in CNS

Recent years, it has been demonstrated that miR-124 is the crucial player to the beginning and development of neurodegenerative diseases, and may be recognized as a biological marker for early identification or as a potential therapeutic target for this kind of diseases (Nguyen et al. 2015; Ambrogini et al. 2018). Most studies have shown that miR-124 expressions are suppressed both in Alzheimer's patients or animal models. Restoring the expression of miR-124 or giving exogenous miR-124 can ameliorate the symptoms of AD (Jin et al. 2013; Woldemichael and Mansuy 2016). Zhang et al. proved that when chronic cerebral hypoperfusion (considered to be a high risk factor for AD) occurred, amyloid β-peptide could inhibit miR-124 and promote the beta-site amyloid precursor protein cleaving enzyme 1(BACE1) expression, which was identified as a rate-limiting enzyme for amyloidβ-peptide synthesis. At the same time, amyloid β-peptide could also inhibit the expression of miR-124 by activating the EPAC-rap1 pathway (Zhang et al. 2017). And recently, it has been found that microvascular dysfunction of nervous system reduces the clearance rate of amyloid β-peptide and promotes the accumulation of amyloid β-peptide in brain tissue. The possible mechanism is that the dysregulation of miR-124 leads to the increase of C1pl3 expression, which reduces micro-vessel density (Li et al. 2019). These mechanisms together form positive feedback to accelerate the progress in AD, and eventually leading to cognitive impairment. Thus, it is miR-124 that has become the key factor of this positive feedback. Experiments confirm that supplementation of exogenous miR-124 can interrupt this process. But Wang et al. research validated that over-expression of miR-124 was detrimental to Alzheimer's patients. They found that the expression of miR-124 was abnormally high in the hippocampal and temporal cortex of Tg2576 mice, and the same expression pattern was found in Alzheimer's patients. Further analysis showed that over-expressed miR-124 could target for down-regulating the PTPN1, and lead to learning and memory impairment in AD model. Memory impairment caused by over-expression of miR-124 can be saved by the application of miR-124 inhibitors or blocking the binding of miR-124 to PTPN1 (Fig. 2) (Wang et al. 2018).

Saraiva found that the differentiation ability of neural stem/progenitor was enhanced after lateral ventricle injection of miR-124 nanoparticles in PD model mice treated with 6-hydroxydopamine (6-OHDA). It was believed that miR-124 could improve the PD symptoms by increasing the number of neurons that migrated into the olfactory bulb and damaged striatum (Saraiva et al. 2016). Wang et al. found that over-expressed miR-24 could effectively reduce the loss of the dopaminergic neurons in the 1-methyl-4-phenyl-1,2,3,6-tetrahydropyridine (MPTP) model mice, and reduce the apoptosis of the dopaminergic neurons by directly down-regulating the activity of the Bim/Bax (Wang et al. 2016). Recently, Yao et al. found that levels of p62/p-p38 (both related to neuroinflammation and autophagy) in midbrain was significantly increased, microglia were activated, while miR-124 level was significantly lower than that of normal, and the autophagy of neurons were inhibited, resulting in apoptosis of neurons in the model of MPTP-induced PD. The suppression of miR-124 and the activation of microglia was consistent with previous studies. Further researches showed that miR-124 exerted as a neuroprotective factor by directly down-regulating the expression of p62/p-p38, promoting neuron autophagy, preventing death and apoptosis of neurons and finally reducing the chronic neuroinflammation. Summarily, it was found that the role of miR-124 in PD was consistent, that was, the decrease of miR-124 was ubiquitous in PD. Exogenous miR-124 or over-expression of miR-124 could improve the PD. Therefore, miR-124 is expected to become a new target for PD.

Nowadays, HD is still considered to be an incurable and life-threatening neurodegenerative disease, and its etiology is generally recognized as abnormal amplification of CAG sequences encoding Huntington's gene. MiR-124 is down-regulated in the striatum of Huntington patients or experimental animals and is thought to be involved in the pathogenesis of HD (Johnson et al. 2008; Packer et al. 2008). As to the repression of miR-124, it was possibly due to the mutant huntingtin (HTT) protein which induced abnormal accumulation of REST in the nucleus and subsequently led to a depression of many non-neuronal genes, changed the identity of neurons, and finally lost neurons. On the other hand, the repression of miR-124 weakened the differentiation of NSPCs, few renewed neurons were produced, but did not satisfy the supplement of damaged neurons, leading to become one of the provital factors for the continuous progress of HD. It is validated that miR-124 can affect the expression of target gene by degrading mRNA or interfering protein synthesis in cytoplasm rather than in nucleus (Drouin-Ouellet et al. 2017). Thus, REST, which is abnormally enriched in nucleus in HD, cannot fully interact with exogenousmiR-124 in cytoplasm, and the downstream non-neuron gene of REST cannot be completely regulated by miR-124. Therefore, the dysfunction of REST or the homeostasis of REST/miR-124 interaction in the nucleus might be recognized as the core factor rather than mere disorder of miR-124 expression. In summary, miR-124 may be the core element of HD pathogenesis, and considered as an important treatment direction in the future (Johnson and Buckley 2009). Therefore, HD animal model was used to evaluate the effect of exogenous miR-124 on HD, and as a result, it showed that exogenous miR-124 could decrease the level of REST protein, but neither increasing in mature neurons nor improvement of motor dysfunction caused by HD could be observed (Lee et al. 2017).

## Conclusion

MiRNAs are still a hot area in the study of brain development and pathology. According to the current researches, it's still very limited for us to deeply understand the mechanism of miRNAs in the development and pathophysiology of central nervous system (CNS). As the most abundant miRNA in the brain, miR-124 makes up a huge and complex neuroregulatory network composed of up- and down-stream regulatory genes, and the network is continually extended and broadened with follow-up studies. This complex regulatory network facilitates us to understand the role of miR-124 in the brain more deeply. The expression of miR-124 is dynamically regulated in different stages of brain development (different temporal and spatial expression patterns). Existing studies have confirmed that miR-124 is involved in the proliferation and differentiation of neural stem cells, determination of cell fate, migration of nerve cells, formation and plasticity of synapses, alteration of cognitive function, repairment of neurons after damage, cell apoptosis and neurodegenerative diseases and all of these reveal many mechanisms of pathophysiological processes in the CNS. It has become a star molecule in the study of brain development and nervous diseases evolution. The specific up- or down-regulation of miR-124 in different diseases has become a new direction for the treatment of nervous system diseases. Therefore, regulating the expression of miR-124 in different developmental stages or different pathological conditions can be used as a very promising treatment, however, more excellent clinical trials need to be carried out.
